# Preliminary Study of Main Pathogenicity Factors and Metabolites of *Wilsonomyces carpophilus*

**DOI:** 10.3390/plants15081202

**Published:** 2026-04-14

**Authors:** Ziyan Xu, Hailong Lu, Chenxu Luo, Chuli Liu, Xinmei Zhou, Rong Ma

**Affiliations:** 1 College of Forestry and Landscape Architecture, Xinjiang Agricultural University, Urumqi 830052, China; m15609933188@163.com (Z.X.); luhailong0908@163.com (H.L.); 13207916673@163.com (C.L.); 15199065502@163.com (C.L.); 13699955465@163.com (X.Z.); 2 State Key Laboratory of Efficient Production of Forest Resources, Beijing Forestry University, Beijing 100083, China

**Keywords:** toxins, cell wall-degrading enzymes, enzyme activity, toxin properties, optimization of toxin production conditions

## Abstract

Shot-hole disease caused by *Wilsonomyces carpophilus* poses a significant threat to stone fruit species, including wild apricot (*Prunus armeniaca* L.). This study investigated pathogenic factors (cell wall-degrading enzymes and toxins) and metabolites produced by a highly pathogenic strain (CFCC 71544) and a weakly pathogenic strain (CFCC 71543) of *W. carpophilus* during infection of *P. armeniaca* (in planta conditions). Analysis using the 3,5-dinitrosalicylic acid colorimetric method revealed that polygalacturonase (CFCC 71544: 1367.02 U/g; CFCC 71543: 1264.00 U/g) and polymethylgalacturonase (CFCC 71544: 1898.71 U·g^−1^; CFCC 71543: 1762.21 U·g^−1^) were the most active cell wall-degrading enzymes, with higher activities observed in the highly pathogenic strain (CFCC 71544). Crude toxins from CFCC 71543 induced leaf lesions averaging 41.91 mm^2^ and retained activity after exposure to 121 °C and UV treatment. Non-protein fractions of the toxins caused significantly larger lesions than protein fractions (15.93 mm^2^ vs. 5.56 mm^2^, respectively). Building on these in planta findings, we further characterized toxin properties under controlled laboratory conditions (in vitro). Optimal toxin production conditions were identified in Richard culture medium at pH 4, under a 12 h light/dark cycle, shaken for 12 days at 25 °C. Untargeted metabolomics identified 3244 compounds and 977 differential metabolites among mycelia, crude toxins, and the residual aqueous phase after organic solvent extraction; these metabolites were predominantly amino acids and derivatives and organic acids. These findings indicate that the main pathogenic factors of *W. carpophilus* are highly active polygalacturonase and heat/UV-stable, water-soluble, non-protein toxins, providing a theoretical basis for shot-hole disease prevention and control.

## 1. Introduction

The fungal shot-hole disease caused by *Wilsonomyces carpophilus* (Lév.) Adask., J.M.Ogawa & E.E.Butler, represents an important pathological threat to the wild fruit forests in Yili, which is in the western Tianshan Mountains of Xinjiang, China [1,2]. Shot-hole disease has been reported in many stone fruit-growing regions worldwide [3,4,5,6,7]. Owing to its extensive geographical distribution and broad host range, *W. carpophilus* poses a significant risk to various stone fruit species, including apricot *(Prunus armeniaca* L.), wild cherry plum (*Prunus cerasifera* Ehrh.), cherry (*Prunus avium* L.), and flat peach (*Prunus persica* var. *compressa* (Loudon) Bean) [8,9]. Shot-hole disease has severely impacted the conservation of germplasm resources in Tianshan wild fruit forests and the cultivation of commercial stone fruit forests. The pathogen has a broad host range within the Rosaceae family, particularly *Prunus* species, including apricot *(Prunus armeniaca* L.), peach (*P. persica*), and cherry (*P. avium*), among others [3,4,5]. It infects multiple aerial parts of the tree, primarily leaves, fruits, and shoots [6,7]. On leaves, initial purple specks enlarge into necrotic lesions that often abscise, creating the characteristic ‘shot-hole’ appearance. Severe infection can lead to premature defoliation, which weakens trees and reduces photosynthetic capacity [8]. Fruit infection causes scabbing and cracking, leading to yield losses that can range from 30% to over 90% in severe cases [9,10]. Shoot and bud infections can cause dieback, further compromising tree structure and future yields. Shot-hole disease has severely impacted the conservation of germplasm resources in the Tianshan wild fruit forests and the productivity of commercial stone fruit orchards. Previous studies have investigated the epidemiology, fungal host spectrum, and genetic diversity of *W. carpophilus* [3,4,5,6,8,10,11,12,13,14,15]. Current studies of *W. carpophilus* are primarily focused on pathogen identification, monitoring, and control strategies.

The three most intensively studied pathogenic factors are enzymes, toxins, and growth-regulating substances [16]. As early as the 1960s, histological studies indicated that plant pathogens produce cell wall-degrading enzymes (CWDEs) that degrade host cell walls, consistent with a necrotrophic lifestyle [17]. In line with this, whole-genome sequencing of *W. carpophilus* later revealed abundant coding genes for CWDEs and secreted proteins [18], providing a molecular basis for its necrotrophic strategy. The genome also encodes protein kinases, which may help the pathogen maintain structural integrity under stress, facilitating host infection. Liu employed the 3,5-dinitrosalicylic acid (DNS) colorimetric method and Bradford method to demonstrate for the first time that *W. carpophilus* produces six types of CWDEs during both in vivo pathogenic processes and in vitro induction [1].

Fungal toxins are pathogenic factors that exert toxic effects on plants [19]. Toxins secreted by different pathogens vary in their composition and roles during the pathogenic process. Highly virulent pathogens often utilize plant toxins as key virulence factors to breach host defense systems and facilitate infection. Abolfazl et al. identified two novel secondary metabolites produced by *W. carpophilus*; however, the pathogenic potential of these metabolites has not yet been investigated [20].

The aim of this study was to better understand the role of CWDEs and toxins in the pathogenic mechanism of *W. carpophilus* by: (i) comparatively analyzing dynamic differences in the activities of six CWDEs during the infection of *P. armeniaca* by highly and weakly virulent strains of *W. carpophilus* through in vivo inoculation experiments; (ii) optimizing the extraction process for crude toxins and systematically characterizing their heat resistance, photostability, and functional components (proteinaceous/non-proteinaceous); and (iii) performing non-targeted metabolomics to determine the metabolite profile characteristics of mycelia, crude toxins, and aqueous extracts.

## 2. Results

### 2.1. Lesion Area of Prunus armeniaca Leaves Infected with Wilsonomyces carpophilus

**Figure 1:**
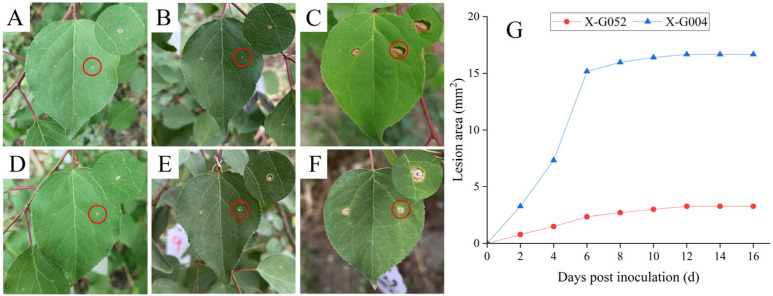
Disease symptoms and lesion expansion rate of *Prunus armeniaca* after inoculation with *Wilsonomyces carpophilus*. (**A**–**C**) Symptoms on leaves 2, 6, and 10 days post-inoculation with strain CFCC 71544, respectively; (**D**–**F**) symptoms on leaves 2, 6, and 10 days post-inoculation with strain CFCC 71543, respectively; (**G**) lesion development after inoculation with strain CFCC 71543 or strain CFCC 71544. X-G052: *P. armeniaca* leaves inoculated with strain CFCC 71543; X-G004: *P. armeniaca* leaves inoculated with strain CFCC 71544.

*P. armeniaca* leaves inoculated with strains CFCC 71544 or CFCC 71543 developed symptoms similar to those observed in the field, whereas control leaves remained symptom-free. Two days after inoculation, circular lesions with white centers were observed (Figure 1A,D). As pathogen infection progressed, mesophyll tissue within approximately 0.5 mm of the lesion margin thinned and showed chlorosis, forming a distinct light-green boundary with healthy tissue (Figure 1B,E). Ten days after boundary formation, lesions stopped expanding and gradually detached from healthy tissue, forming shot holes (Figure 1C,F). Lesions produced by the weakly virulent strain CFCC 71543 (3.27 mm^2^, n = 6) were significantly smaller than those produced by the highly virulent strain CFCC 71544 (16.66 mm^2^, n = 6) (*p* < 0.05, Duncan’s multiple range test). (Figure 1G).

### 2.2. Changes in the Activity of Cell Wall-Degrading Enzymes in Prunus armeniaca Infected with Wilsonomyces carpophilus

**Figure 2:**
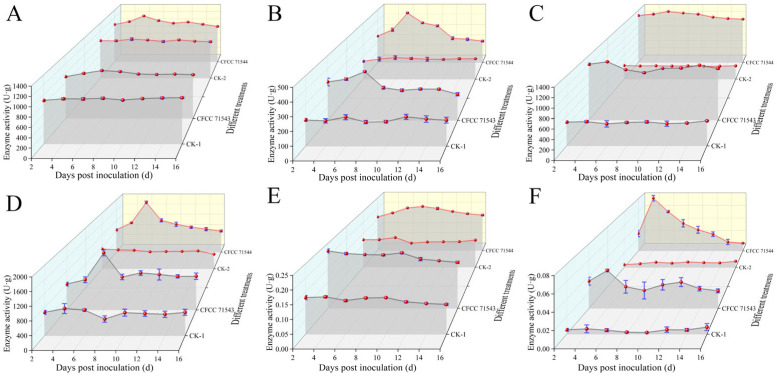
Changes in the activity of cell wall-degrading enzymes during pathogenesis. (**A**) Carboxymethyl cellulase; (**B**) β-glucosidase; (**C**) polygalacturonase; (**D**) polymethylgalacturonase; (**E**) polygalacturonate transeliminase; (**F**) pectin methylesterase transeliminase. CK1, control 1 (mock-inoculated leaves of *Prunus armeniaca* trees); CFCC 71543, leaves of *P. armeniaca* trees inoculated with *Wilsonomyces carpophilus* strain CFCC 71543; CK2, control 2 (mock-inoculated leaves of *P. armeniaca* trees); CFCC 71544, leaves of *P. armeniaca* trees inoculated with *W. carpophilus* strain CFCC 71544.

Activities of six CWDEs in *P. armeniaca* leaves markedly increased following inoculation with either the weakly virulent CFCC 71543 strain or the highly virulent CFCC 71544 strain, with peak values predominantly observed 4–6 days post-inoculation (dpi). Carboxymethyl cellulase activity in leaves infected with CFCC 71543 or CFCC 71544 was 1.16-fold and 1.26-fold higher than the control, respectively (maximum levels of 1055.80 U·g^−1^ and 1244.70 U·g^−1^, respectively, before gradually declining (Figure 2A). β-glucosidase activity surged rapidly between 2 and 6 dpi, peaking at 375.47 U·g^−1^ (CFCC 71543) and 471.38 U·g^−1^ (CFCC 71544) at 6 dpi (1.82-fold and 2.45-fold higher than the control, respectively; Figure 2B). The peak values for PG activity were strain-specific, with CFCC 71543 infection resulting in a peak of 1264.00 U·g^−1^ at 4 dpi (2.61-fold higher than the control) and CFCC 71544 infection producing a peak of 1367.02 U·g^−1^ at 6 dpi (4.09-fold higher than the control; Figure 2C). PMG activity increased sharply between 2 and 6 dpi, peaking at 1762.21 U·g^−1^ (CFCC 71543) and 1898.71 U·g^−1^ (CFCC 71544) at 6 dpi (2.42-fold and 2.86-fold higher than the control, respectively; Figure 2D). PGTE dynamics differed between strains, with CFCC 71543 infection resulting in an initial peak of 0.22 U·g^−1^ at 2 dpi (1.7-fold higher than the control) and a second peak at 10 dpi, whereas CFCC 71544 infection resulted in a continuous increase from 2–6 dpi, peaking at 0.21 U·g^−1^ at 8 dpi (1.94-fold higher than the control; Figure 2E). PMTE activity spiked between 2 and 4 dpi, reaching maxima of 0.047 U·g^−1^ (CFCC 71543) and 0.081 U·g^−1^ (CFCC 71544) at 4 dpi, 7.83-fold and 16.2-fold higher than the control, respectively (Figure 2F).

### 2.3. Basic Properties of Crude Toxins Produced by Wilsonomyces carpophilus and Optimal Toxin Production Conditions

**Figure 3:**
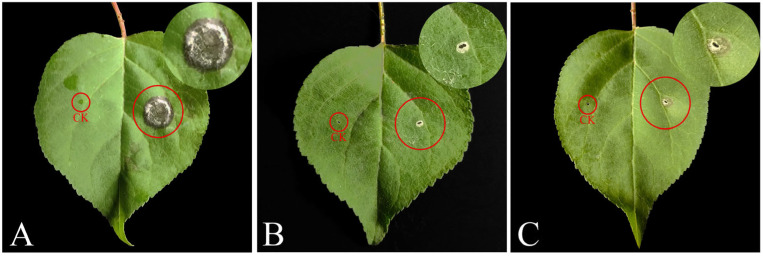
Wounded *Prunus armeniaca* leaves applied with toxins or fractions extracted from *Wilsonomyces carpophilus*. (**A**) Crude toxin stock solution; (**B**) protein fraction; (**C**) non-protein fraction. CK, control.

**Figure 4:**
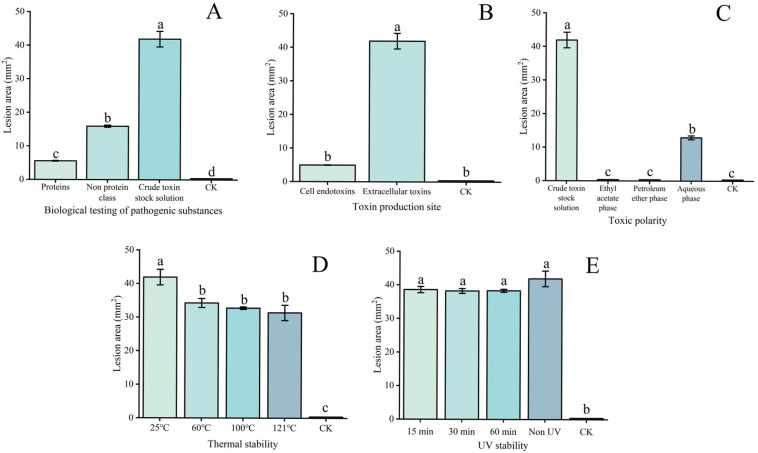
Bioassays to determine the basic properties of crude toxins produced by *Wilsonomyces carpophilus* strain CFCC 71543 based on the lesion area that developed following the application of crude toxins to wounded *Prunus armeniaca* leaves. (**A**) Pathogenic substances; (**B**) toxin production site; (**C**) toxin polarity; (**D**) thermal stability of toxins; (**E**) UV stability of toxins. Different lowercase letters in the same column indicate a significant difference at the *p* < 0.05 level, as determined by Duncan’s new multiple range test.

The application of crude toxin stock solution to *P. armeniaca* leaf wounds resulted in the development of a significantly larger mean lesion area (41.91 mm^2^) than the application of protein and non-protein fractions (5.56 mm^2^ and 15.93 mm^2^, respectively), as well as chlorosis and rot symptoms (Figure 3 and Figure 4A). The application of the non-protein fraction also caused chlorosis and rot (Figure 3C), whereas the protein fraction only resulted in mild whitening of tissues at the leaf wound sites (Figure 3B), indicating that non-protein substances are the key toxin components responsible for the virulence of *W. carpophilus*.

The application of extracellular toxins to wounded leaves led to the development of significantly larger lesions than the application of intracellular toxins (41.91 mm^2^ vs. 4.93 mm^2^, Figure 4B). Polarity testing showed that the application of the aqueous phase fraction led to the development of lesions that were 12.90 mm^2^ (Figure 4C), whereas the application of ethyl acetate or petroleum ether phases led to negligible lesion development.

The thermal stability experiment demonstrated that the activity of the crude toxin stock solution was slightly affected by high temperatures. Leaf wounds applied with toxins subjected to temperatures of 60 °C, 100 °C, or 121 °C developed lesions of 31.36 mm^2^ compared with lesions of 41.91 mm^2^ when applied with toxins cultured at 25 °C (Figure 4D). Crude toxins were exposed to UV irradiation for 15, 30, or 60 min while being maintained at a constant temperature of 25 °C. UV tolerance tests showed that leaf wounds applied with a crude toxin stock solution that were subjected to UV irradiation for up to 60 min developed lesions that were not significantly different from those that developed following the application of a non-irradiated crude toxin stock solution (38.32 mm^2^ vs. 41.91 mm^2^, respectively; Figure 4E).

**Figure 5:**
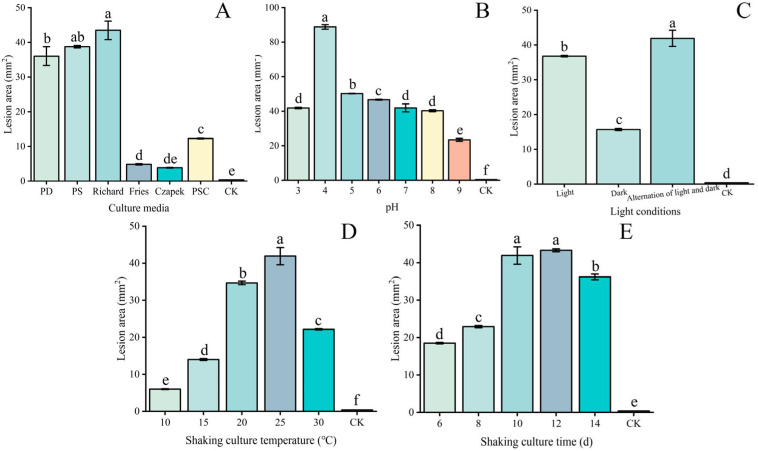
Screens to determine the optimal culture conditions for toxin production by *Wilsonomyces carpophilus* based on the lesion area that developed on *Prunus armeniaca* leaves following the application of crude toxins. (**A**) Culture media (PD, potato dextrose; PS, potato sucrose; PSC, potato sucrose culture); (**B**) pH values; (**C**) light conditions; (**D**) shaking culture temperature; (**E**) shaking culture time. For panel E, cultures were grown in Richard medium at pH 4, 25 °C, under a 12 h light/dark cycle with shaking and harvested at the indicated times. Different lowercase letters in the same column indicate a significant difference at the *p* < 0.05 level, as determined by Duncan’s new multiple range test.

Significantly larger lesion areas (43.48 mm^2^) developed in leaves applied with crude toxins produced by *W. carpophilus* CFCC 71543 grown in Richard’s medium than when grown in other media, confirming Richard’s medium as the optimal culture substrate for toxin production (Figure 5A). Furthermore, toxin activity peaked using Richard’s medium adjusted to pH 4, with significantly larger lesion areas (88.81 mm^2^) developing than at other pH levels (Figure 5B). A 12 h light/dark cycle was the most favorable light pattern for toxin accumulation, resulting in the development of lesion areas of 41.91 mm^2^, 2.7-fold larger than those that developed when toxins were produced under continuous dark conditions (Figure 5C). The optimal temperature for toxin production was 25 °C, resulting in the development of lesion areas that were approximately three times larger than those produced by toxins extracted from cultures incubated at 15 °C (41.91 mm^2^ vs. 13.99 mm^2^; Figure 5D); a temperature of 30 °C also significantly inhibited toxin biosynthesis. As shown by the shaking culture time, when leaves were inoculated with crude toxin collected on day 6 of culture, the mean lesion area was 18.483 mm^2^. As culture time progressed, lesion area increased continuously, reaching 22.893 mm^2^ to 41.910 mm^2^ between day 8 and day 10. By day 12 of culture, toxin production reached its peak, with crude toxin from this time point producing a mean lesion area of 43.294 mm^2^. However, when culture continued beyond 12 days, the pathogenic effect of the toxin began to decline, with the lesion area gradually decreasing to 36.197 mm^2^ by day 16. These results demonstrate that toxin production by *Wilsonomyces carpophilus* follows a temporal pattern, with maximum activity observed at 12 days of culture under the conditions tested (Figure 5E).

### 2.4. Metabolomics Study of Wilsonomyces carpophilus Mycelia and Crude Toxins

#### 2.4.1. Liquid Chromatography–Mass Spectrometry-Based Metabolite Analysis

A non-targeted metabolomics approach using liquid chromatography–mass spectrometry (LC-MS) was employed to analyze metabolites in six biological samples of *W. carpophilus*, including mycelia, crude toxin stock solution, and the residual aqueous phase after organic solvent extraction. A total of 3244 metabolites were detected across all samples (Table 1).

**Figure 6:**
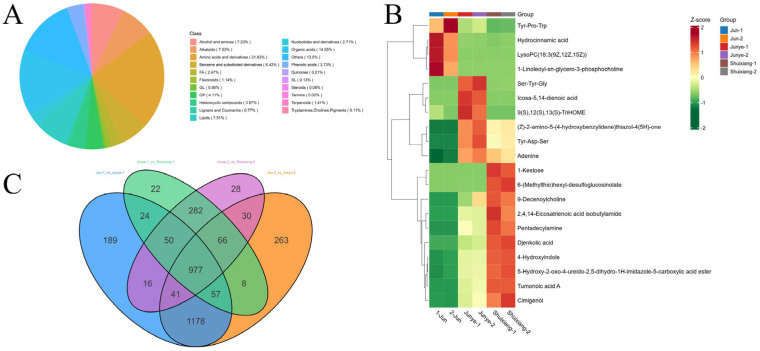
Metabolic analysis of toxin from *Wilsonomyces carpophilus*. (**A**) Proportion of each primary metabolite class detected in the total metabolite pool of *Wilsonomyces carpophilus*; (**B**) cluster heatmap of a differential analysis of the primary metabolic contents of mycelia, crude toxin stock solution, and the aqueous phase of *Wilsonomyces carpophilus*; (**C**) Venn diagram of common differential metabolites in different fractions of *Wilsonomyces carpophilus* culture broth. Note: Jun-1 and Jun-2: These represent two biological replicates of the mycelia samples collected from *Wilsonomyces carpophilus* cultures. Junye-1 and Junye-2: These represent two biological replicates of the crude toxin stock solution (culture filtrate) samples. Shuixiang-1 and Shuixiang-2: These represent two biological replicates of the aqueous phase samples obtained after organic solvent extraction of the crude toxin.

Most major metabolites were present in the mycelia, crude toxin stock solution, and residual aqueous phase after extraction of *W. carpophilus*: amino acids and derivatives, organic acids, alkaloids, lipids, alcohols and amines, and benzene and substituted derivatives were the dominant metabolites (Figure 6A).

#### 2.4.2. Differential Metabolite Cluster Analysis

The major metabolites found in mycelia, crude toxin stock solution, and the extracted aqueous phase of *W. carpophilus* differed significantly. The most abundant metabolites in the mycelia included: Tyr-Pro-Trp (amino acids and derivatives), hydrocinnamic acid (phenolic acids), lysoPC (18:3 (9Z, 12Z, 15Z)) (lipids), and 1-linoleoyl-sn-glycero-3-phosphocholine (glycerophospholipids). In the crude toxin stock solution, the dominant metabolites were Ser-Tyr-Gly (amino acids and derivatives), icosa-5,14-dienoic acid (organic acids), 9(S),12(S),13(S)-TriHOME (lipids), (Z)-2-amino-5-(4-hydroxybenzylidene) thiazol-4 (5H)-one (benzene and substituted derivatives), Tyr-Asp-Ser (amino acids and derivatives), and adenine (nucleotides and derivatives). The extracted aqueous phase of the crude toxin stock solution featured primary metabolites such as: 1-kestose (carbohydrates), 6-(methylthio)hexyl-desulfoglucosinolate (lactones), 9-decenoylcholine (lactones), 2,4,14-eicosatrienoic acid isobutylamide (fatty acyls), pentadecylamine (alcohols and amines), djenkolic acid (organic acids), 4-hydroxyindole (alkaloids), tumonoic acid A (organic acids), cimigenol (alcohols and amines), and 5-hydroxy-2-oxo-4-ureido-2,5-dihydro-1H-imidazole-5-carboxylic acid ester (organic acids) (Figure 6B).

#### 2.4.3. Identification of Common Differential Metabolites in Different Fractions of *Wilsonomyces carpophilus* Crude Toxin Stock Solution

A Venn diagram analysis revealed 977 differential metabolites that were present in mycelia, the crude toxin stock solution, and the aqueous phase, including 293 amino acids and derivatives, 87 benzene and substituted derivatives, 52 alcohols and amines, 33 phenolic acids, 20 glycerophospholipids, 5 glycerolipids, 35 nucleotides and derivatives, 13 flavonoids, 1 quinone, 6 lignans and coumarins, 1 tannin, 59 alkaloids, 27 terpenoids, 131 organic acids, 35 heterocyclic compounds, 1 steroid, 9 fatty acyls, 50 lipids, and 1 other (Figure 6C).

## 3. Discussion

During the invasion of host plants, plant pathogenic microorganisms first disrupt the cell wall structure and related tissues by producing CWDEs, which accelerate pathogen invasion, nutrient absorption, and parasitic proliferation, ultimately leading to disease development [21]. In a study of apple anthracnose, Xue found that PMG and Cx played key roles in the infection of apples by *Colletotrichum gloeosporioides* [22]. However, Gai et al. identified PG and Cx as the critical enzymes involved in the infection of citrus by *C. gloeosporioides*, indicating that the primary CWDEs used by a pathogen may differ when infecting different hosts [23]. During infection of *P. armeniaca* by *W. carpophilus* strains with different pathogenic abilities, the activity trends of the six CWDEs analyzed in this study were consistent, and PG was the main CWDE. By contrast, Lu et al. reported that PMG was the primary CWDE produced by *W. carpophilus* when infecting *Prunus davidiana* (Carrière) Franch [24] (David’s peach), supporting the idea that the major CWDEs produced by *W. carpophilus* may differ among different host species. The activities of the six CWDEs produced by the strongly pathogenic strain (CFCC 71544) were significantly higher than those of the weakly pathogenic strain (CFCC 71543) throughout the infection process. However, we only explored the activities of six CWDEs. Whether other CWDEs such as laccase are produced during pathogenesis requires further investigation.

Fungi produce a diverse range of metabolites in terms of their chemical structures and biological properties, and, therefore, a range of different methods need to be used to extract crude toxins from culture broth [25]. The production of extracellular toxins by pathogenic fungi is influenced by factors such as culture medium composition and environmental conditions. The relationship between culture medium (including components such as carbon sources, nitrogen sources, mineral nutrients, and natural organic matter) and toxin production is extremely close [26]. Our analyses of different fractions of the pathogen culture broth revealed that the primary toxins produced by *W. carpophilus* are non-protein substances. Characterization of these non-protein toxins revealed that they exhibit high polarity—they cannot be effectively extracted using organic solvents—and have high thermal stability and UV light resistance. Our analyses also demonstrated that different types of fermentation media affect the growth of the pathogen. Temperature is a critical factor that affects mycelial growth rates, with excessively low temperatures slowing mycelial growth and, thus, reducing the toxin production capacity of the pathogen. The toxin production capacity of the pathogen increases gradually with culture duration but declines after reaching a peak. Extremely acidic or alkaline conditions, as well as specific light regimes, also act as major inhibitors of mycelial growth.

This study systematically characterized the metabolic profiles of *W. carpophilus* mycelia, crude toxins, and aqueous extraction phase using non-targeted metabolomics. A total of 3244 metabolites were identified across the tested samples, with amino acids and derivatives (28.11%) and organic acids (10.60%) being the most abundant—these chemical groups are closely linked to the accumulation of precursor substances in fungal toxin biosynthesis. The presence of 977 differential metabolites that were found in mycelia, the crude toxin stock solution, and the aqueous phase supports the hypothesis that the pathogenic functions of *W. carpophilus* are achieved through the dynamic regulation of its metabolic network during infection. Mycelia were dominated by metabolites such as Tyr-Pro-Trp (amino acid derivative) and hydrocinnamic acid (phenolic acid), whereas the crude toxin stock solution was dominated by Ser-Tyr-Gly and Icosa-5,14-dienoic acid (organic acid). This differential metabolite distribution likely reflects functional specialization in different pathogen compartments: phenolic acids in mycelia may facilitate host cell wall penetration, whereas the excretion of organic acids detected in the crude toxin stock solution could enhance toxin activity by acidifying the microenvironment. In addition, the high abundance of non-protein fractions detected in crude toxins (e.g., djenkolic acid and tumonoic acid A) is consistent with our observation that cultures subjected to 121 °C and UV treatment maintained their pathogenicity, supporting the conclusion that non-protein toxins play a crucial role in pathogenesis.

The invasion of host plants by pathogenic fungi is a complex process involving not only the secretion of CWDEs and toxins but also the production of growth regulators such as hormones. Given the complexity of pathogenic mechanisms during the infection process, how different pathogenic factors coordinate their actions—such as the interplay between CWDEs, toxins, and signaling molecules—remains to be further investigated. A limitation of this study is the lack of clarification regarding the specific functional pathways of differential metabolites and their causal relationship with pathogenic phenotypes. Future research should integrate transcriptomics and gene knockout techniques to screen target genes regulating key metabolite synthesis, providing a theoretical basis for developing novel control strategies based on metabolic interference.

## 4. Materials and Methods

### 4.1. Tested Strains and Host Plants

A highly virulent strain (CFCC 71544) and a weakly virulent strain (CFCC 71543) of *Wilsonomyces carpophilus* were used in this study [9]. The host plants were *Prunus armeniaca* trees growing in an experimental field of Xinjiang Agricultural University that were more than 5 years old.

### 4.2. Inoculation of Prunus armeniaca and Measurement of the Lesion Area

Healthy *P. armeniaca* leaves with uniform physiological status and no mechanical damage or pest infestation were selected as experimental materials, following a previously published protocol [27,28]. For each treatment (CFCC 71544, CFCC 71543, and control), three individual plants were used, with one leaf selected from each plant to serve as a biological replicate. Leaf surfaces were surface-sterilized with 75% ethanol for 30 s, rinsed three times with sterile water to remove residual alcohol, and then allowed to air-dry naturally. A sterilized insect needle (diameter = 0.5 mm) was used to create two wounds in non-venous regions of each inoculated leaf, with one wound placed on each side of the main vein. A mycelial plug (5 mm diameter) from the edge of 7-day-old cultures of strain CFCC 71544 or strain CFCC 71543 growing on potato dextrose agar (PDA) medium was precisely placed onto each wound with the mycelial side in contact with the wound surface. Local humidity was maintained by placing moistened absorbent cotton over the wound and sealing it with Parafilm to retain moisture, creating a humid microenvironment conducive to infection. The moistened absorbent cotton was removed from wounds 24 h post-inoculation. Inoculation sites were sealed with Parafilm to retain moisture. Wounded leaves of control plants were mock-inoculated with sterile PDA plugs. The moistened absorbent cotton was removed from wounds 24 h post-inoculation. From 48 h post-inoculation onwards, lesion lengths and widths were measured using a vernier caliper (precision 0.1 mm) every 48 h. All six inoculation sites per strain (three leaves × two wounds per leaf) were measured and included in the statistical analysis.

### 4.3. Extraction of Cell Wall-Degrading Enzymes

Wild apricot leaves inoculated as described in Section 4.2. were sampled at 2, 4, 6, 8, 10, 12, 14, and 16 days post-inoculation (dpi). Leaf tissue (100 mg) at the junction between diseased and healthy tissue was excised, placed into a homogenizer containing 3 mL of extraction buffer (20 mM Tris-HCl, pH 7.4, containing 1 M NaCl), and homogenized on ice for 2 min. An additional 7 mL of the same extraction buffer was then added, and the mixture was centrifuged at 10,000 rpm for 15 min at 4 °C. The supernatant (crude enzyme extract) was collected and then stored at 4 °C until ready for use in subsequent assays [29].

### 4.4. Preparation of Standard Curves

Glucose standard curves were prepared using glucose solutions at different concentrations and DNS reagent, yielding a regression equation of y = 2.294x + 0.0195 (R^2^ = 0.9965). D-galacturonic acid standard curves were constructed using D-galacturonic acid solutions and DNS reagent, with the regression equation y = 2.5886x + 0.0188 (R^2^ = 0.9941). BSA standard curves were generated based on OD_595_ values of BSA solutions of different concentrations reacted with Coomassie Brilliant Blue G250, resulting in the regression equation y = 0.0008x + 0.0053 (R^2^ = 0.9923).

A 100 mL glucose standard solution (1 mg·mL^−1^) was prepared with distilled water. Different volumes of the glucose solution, distilled water, and 3,5-dinitrosalicylic acid (DNS) reagent were mixed thoroughly and then placed in a boiling water bath for 5 min. After cooling to room temperature and shaking thoroughly, the OD_540_ values of each tube were measured using a spectrophotometer. A standard curve was constructed with glucose concentration on the x-axis and OD_540_ values on the y-axis. Standard curves for D-galacturonic acid and bovine serum albumin (BSA) were prepared using the same procedure [29,30].

### 4.5. Activity Assays of Cell Wall-Degrading Enzymes

Enzyme activities of carboxymethyl cellulase (Cx), β-glucosidase, polymethylgalacturonase (PMG), PG, polygalacturonate transeliminase (PGTE), and pectin methylesterase transeliminase (PMTE) were determined following the methods described by Douaiher et al. and Siah et al. [31,32]. Using the DNS colorimetric method, the absorbance value of each reaction mixture was measured at 540 nm with a spectrophotometer, and the enzyme activity was calculated based on the amount of reducing sugars released by enzymatic reactions. Enzyme activity units were defined as the amount of enzyme required to catalyze the production of 1 μM of reducing sugar per min per mL of enzyme solution (or per mg of protein) at 50 °C, with the amount of reducing sugar generated quantified using standard curves.

### 4.6. Preparation of Crude Toxins

PDA plates were inoculated with strain CFCC 71543 and incubated at 25 °C in an incubator for 7 days. Five mycelial plugs (5 mm inner diameter, obtained using a cork borer) were transferred to a flask containing 100 mL of modified Richard’s liquid medium and cultured on a horizontal shaker at 150 revolutions per minute (rpm) at 25 °C for 12 days. The culture broth was filtered through two layers of filter paper to separate mycelia, followed by vacuum filtration to obtain a clear liquid, which served as the crude toxin stock solution [33].

### 4.7. Investigation of the Basic Properties of Crude Toxins

First, protein and non-protein components in the crude toxin stock solution were separated by methanol precipitation: 200 mL of methanol was added to the solution, followed by centrifugation at 8000 rpm for 20 min at 4 °C to achieve phase separation. The protein fraction was redissolved in distilled water to return it to the original volume, whereas the non-protein fraction was concentrated by rotary evaporation at 40 °C to remove methanol and then redissolved in sterile water to return it to the same volume. The biological activities of both fractions were assayed separately. To investigate extracellular toxin production, a double filtration was performed. This involved filtering the culture broth through two layers of filter paper, followed by vacuum filtration. Intracellular toxins were obtained by disrupting mycelial cell walls using a bead mill, extracting metabolites with 200 mL of methanol over a period of 3 days, and then redissolving the filtrate in 100 mL of ultrapure water. The bioactivities of toxins were compared synchronously. For toxin polarity analysis, a gradient extraction strategy was adopted: toxins were sequentially extracted three times from the crude toxin stock solution using equal volumes of petroleum ether (shaken at 200 rpm for 30 min at 25 °C) and ethyl acetate. After phase separation using a separating funnel, the petroleum ether phase, ethyl acetate phase, and aqueous phase were collected, concentrated (petroleum ether phase, 0.1 MPa, 45 °C; ethyl acetate phase, 0.1 MPa, 45 °C), and their activities were measured. To assess the stability of toxins in the environment, the crude toxin solution was subjected to temperatures of 25 °C (control), 60 °C, 100 °C, or 121 °C for 20 min and exposed to UV irradiation for 0, 15, 30, or 60 min. All biological activity assays followed a standardized procedure: wild apricot (*P. armeniaca*) leaves were pricked with an alcohol-sterilized insect needle (Φ = 0.5 mm) and 100 μL of the test solution was applied to the wound, with three biological replicates per treatment to ensure data reliability. After 96 h of incubation under humid conditions, the lesion area was measured for each replicate, respectively [25].

### 4.8. Optimization of Toxin Production Conditions

A multidimensional gradient screening strategy was used to determine the optimal culture conditions for toxin production by strain CFCC 71543. First, six different liquid media—Richard, modified Czapek, potato sucrose culture, potato dextrose, Fries, and potato sucrose—were inoculated with strain CFCC 71543 and shaken for 12 days at 25 °C. Crude toxin solutions were extracted and their activities compared to determine the optimal medium for toxin production. Based on these results, the optimal pH for toxin production was evaluated by adjusting Richard’s medium to different pH levels (pH 3–9), with HCl/NaOH and shaking at 25 °C for 12 days. With the optimal medium (Richard) and pH (pH 4) determined, the optimal environmental parameters for toxin production were sequentially determined by assessing different light conditions (i.e., continuous light, continuous dark, and 12 h light/dark cycles), shaking temperatures (i.e., 10, 15, 20, 25, and 30 °C), and shaking periods (i.e., 6, 8, 10, 12, and 14 days). All cultures were incubated at 25 °C except during the temperature optimization experiment. All experimental groups were validated using a standardized bioactivity assay procedure: *P. armeniaca* leaves were pricked with an alcohol-sterilized insect needle (Φ = 0.5 mm), 100 μL of the test solution was applied to the wound, with three biological replicates per treatment to ensure data reliability [34,35]. After 96 h of incubation in a humid environment, the lesion area was quantitatively analyzed.

### 4.9. Non-Targeted Metabolomics Analysis

Three types of biological sample were subjected to metabolomics analysis: (1) *W. carpophilus* mycelia (from the vacuum filtration residue described in Section 4.6); (2) crude toxin stock solution (from the filtrate obtained in Section 4.6); and (3) the aqueous extraction phase (from the residual phase after organic solvent extraction in Section 4.7.). These biological samples were subjected to a standardized pretreatment, which was performed sequentially: samples were lyophilized in a Scientz-100F (Ningbo Xinzhi Biotechnology Co., Ltd., Ningbo, China) freeze-dryer for 63 h under a vacuum, crushed to form a powder using a Retsch MM400 (RETSCH, Haan, Germany) grinder (30 Hz, 1.5 min), and 50 mg of this powder (weighed with an MS105DΜ electronic balance) was precisely mixed with 1200 μL of a pre-cooled (−20 °C) 70% methanol internal standard extraction solution. The internal standard solution was prepared via gradient dilution: 1 mg of standard was dissolved in 1 mL of 70% methanol to obtain a 1000 μg·mL^−1^ stock solution, which was further diluted to 250 μg·mL^−1^. After subjecting the mixture to six 30 s vortexing cycles with 30 min intervals between cycles, the mixture was centrifuged at 12,000 rpm for 3 min. The supernatant was filtered through a 0.22 μm microfiltration membrane and transferred to an injection vial. Analysis was conducted using a UPLC-MS/MS system with the following chromatographic conditions: a Waters ACQUITY Premier HSS T3 (Waters, Milford, MA, USA) column (1.8 μm, 2.1 × 100 mm), mobile phases A (0.1% formic acid in water)/B (0.1% formic acid in acetonitrile), column temperature 40 °C, flow rate 0.4 mL·min^−1^, and injection volume 4 μL. This protocol ensures reproducibility and comparability of metabolomics data through precise instrument integration and parameter optimization.

### 4.10. Data Processing

All measured data were processed and analyzed using Excel 2019 to calculate the mean values of three replicates. Analysis of variance was performed using IBM SPSS Statistics 22.0, followed by Duncan’s multiple range test for multiple comparisons and significance analysis of differences. Graphs and charts were created using Origin 2022 (version 9.9) 64-bit software.

Raw mass spectrometry data were converted to mzXML format using ProteoWizard. Peak extraction, alignment, and retention time correction were performed using the XCMS program. Peaks with a missing rate of >50% across samples were filtered out, and blank values were imputed using k-nearest neighbor imputation. Peak areas were corrected using support vector regression. The corrected and filtered peaks were identified by searching an in-house database, integrating public databases (METLIN, KEGG) and prediction databases, and employing the metDNA approach for metabolite annotation.

## 5. Conclusions

This study analyzed six CWDEs—Cx, β-glucosidase, PG, PMG, PGTE, and PMTE—produced by weakly and highly pathogenic strains of *W. carpophilus* during the infection of *P. armeniaca*. Although the primary enzymes remained consistent across strains with different pathogenicity, the timing of peak enzyme activity differed, and the strongly pathogenic strain exhibited significantly higher enzyme activities. Extracellular toxins were the main toxin components produced by strain CFCC 71543 and were characterized by high thermal and UV stability, high polarity, and high water solubility. These toxins included both protein and non-protein fractions, with non-protein toxins dominating. The optimal conditions for culturing *W. carpophilus* for optimal toxin production were Richard’s medium at pH 4, a 12 h light/dark cycle, and shaking for 12 days at 25 °C. A total of 3244 compounds were identified in mycelia, the crude toxin stock solution, and the aqueous extraction phase, including 977 differential metabolites that were common to all three. Both the major and differential metabolites were predominantly amino acids and derivatives and organic acids.

## Figures and Tables

**Table 1:** Number of primary metabolites in *Wilsonomyces carpophilus* mycelia, crude toxin stock solution, and aqueous extraction phase.

Serial No.	Category	Mycelia	Crude Toxin Stock Solution	Aqueous Phase
1	Amino acids and derivatives	912	757	702
2	Benzene and substituted derivatives	348	325	309
3	Alcohols and amines	139	167	159
4	Phenolic acids	97	91	80
5	Glycerophospholipids	120	67	54
6	Glycerolipids	39	25	22
7	Nucleotides and derivatives	76	91	88
8	Flavonoids	63	57	57
9	Quinones	5	5	5
10	Lignans and coumarins	46	18	17
11	Sphingolipids	7	7	6
12	Tannins	3	3	3
13	Tryptamines, cholines, and pigments	7	6	5
14	Alkaloids	171	142	127
15	Terpenoids	83	69	59
16	Organic acids	344	341	320
17	Heterocyclic compounds	118	108	101
18	Steroids	15	5	6
19	Fatty acids	45	46	43
20	Lipids	162	130	123
21	Others	393	400	379
	Total	3193	2860	2665

## Data Availability

All data are included in the paper.

## Abbreviations

The following abbreviations are used in this manuscript:

CWDEs	Cell wall-degrading enzymes
Cx	Carboxymethyl cellulase
PMTE	Pectin methylesterase transeliminase
PG	Polygalacturonase
PMG	Polymethylgalacturonase
PGTE	Polygalacturonate transeliminase
